# The novel imaging methods in diagnosis and assessment of cerebrovascular diseases: an overview

**DOI:** 10.3389/fmed.2024.1269742

**Published:** 2024-04-10

**Authors:** Fei Liu, Ying Yao, Bingcheng Zhu, Yue Yu, Reng Ren, Yinghong Hu

**Affiliations:** ^1^Neuroscience Intensive Care Unit, The Second Affiliated Hospital of Zhejiang University School of Medicine, Hangzhou, Zhejiang, China; ^2^Department of Radiology, The Second Affiliated Hospital of Zhejiang University School of Medicine, Hangzhou, Zhejiang, China

**Keywords:** cerebrovascular diseases, neuroimaging, imaging techniques, diagnosis, assessment

## Abstract

Cerebrovascular diseases, including ischemic strokes, hemorrhagic strokes, and vascular malformations, are major causes of morbidity and mortality worldwide. The advancements in neuroimaging techniques have revolutionized the field of cerebrovascular disease diagnosis and assessment. This comprehensive review aims to provide a detailed analysis of the novel imaging methods used in the diagnosis and assessment of cerebrovascular diseases. We discuss the applications of various imaging modalities, such as computed tomography (CT), magnetic resonance imaging (MRI), positron emission tomography (PET), and angiography, highlighting their strengths and limitations. Furthermore, we delve into the emerging imaging techniques, including perfusion imaging, diffusion tensor imaging (DTI), and molecular imaging, exploring their potential contributions to the field. Understanding these novel imaging methods is necessary for accurate diagnosis, effective treatment planning, and monitoring the progression of cerebrovascular diseases.

## 1 Introduction

Cerebrovascular diseases encompass a diverse range of conditions that affect the blood vessels in the brain and cause damage to brain tissue due to intracranial blood circulation disorders. These conditions mainly refer to ischemic stroke, intracerebral hemorrhage, transient ischemic attack, subarachnoid hemorrhage, subarachnoid hemorrhage and others. The epidemiological studies revealed that more than 62% of new strokes, 69.8% of prevalent strokes, and 45.5% of deaths from stroke because of stroke were in people younger than 75 years, which further illustrated the syndrome of high morbidity, high mortality, and high recurrence (1). Besides, the huge economic burden of diseases continues to be a challenge worldwide. On a positive note, more and more researchers are working on the etiology, pathogenesis, diagnosis and treatment of cerebrovascular diseases and large quantities of remarkable achievements have been made. Among them, novel imaging methods have improved the understanding of the underlying pathophysiology and revolutionized the precise diagnosis, risk stratification and prognosis assessment of cerebrovascular diseases (2).

The emergence of advanced imaging techniques like Magnetic Resonance Imaging (MRI) and Computed Tomography (CT) has greatly improved the diagnostic capabilities in cerebrovascular diseases. These techniques provide detailed anatomical information about the brain and blood vessels, allowing for the identification of abnormalities such as aneurysms, arteriovenous malformations (AVMs), stenosis and so on. Moreover, functional imaging techniques like Functional MRI (fMRI) and Perfusion Imaging offer insights into the brain’s functional activity and blood flow patterns, helping clinicians understand the impact of cerebrovascular diseases on brain function. These techniques have become invaluable in assessing the extent of ischemic brain damage, identifying viable brain tissue, and predicting functional outcomes. In addition to these well-established imaging methods, novel techniques such as Diffusion Tensor Imaging (DTI) and Positron Emission Tomography (PET) have emerged as valuable assets in diagnosing and assessing cerebrovascular conditions (3). DTI enables the evaluation of white matter integrity and connectivity, providing information about the structural changes in cerebrovascular diseases (4). This review aims to explore the novel imaging methods that have emerged as valuable assets in diagnosing and assessing various cerebrovascular conditions.

### 1.1 The role of neuroimaging in cerebrovascular disease management

Neuroimaging is of great significance in the management of cerebrovascular diseases. It provides vital information about the underlying cause, extent, and severity of brain damage. Additionally, neuroimaging techniques aid in assessing the risk of future strokes and guiding appropriate treatment strategies. First of all, CT and MRI are the primary imaging modalities used to evaluate cerebrovascular diseases. CT scans are commonly performed in the acute setting to assess for hemorrhage quickly, identify potential causes of stroke, and rule out other differential diagnoses (5). Conversely, MRI provides superior anatomical detail and is particularly useful in detecting ischemic lesions (6). In addition to structural imaging, advanced neuroimaging techniques can provide valuable functional and physiological information (7, 8). Perfusion imaging techniques, such as CT perfusion and MR perfusion, help assess the blood flow to the brain and identify regions of hypoperfusion (9–11). This information is pivotal in determining the extent of viable tissue and guiding reperfusion therapies, such as thrombolytic therapy or mechanical thrombectomy, in the management of acute ischemic strokes. Furthermore, neuroimaging can aid in the identification of the underlying vascular pathology and guide preventive measures (12, 13). Angiographic techniques, such as CT angiography and magnetic resonance angiography, allow visualization of the brain’s blood vessels and help identify stenosis, occlusions, or aneurysms (14, 15). This information is invaluable in selecting patients who may benefit from surgical or endovascular interventions, like carotid endarterectomy or coil embolization (16, 17).

### 1.2 Importance of novel imaging methods for diagnosis and assessment

Emerging imaging methods are continuously improving our understanding and management of cerebrovascular diseases. One such technique is perfusion-weighted imaging (PWI) using arterial spin labeling, which allows quantitative assessment of cerebral blood flow without the need for exogenous contrast agents. PWI has shown promise in detecting subtle changes in cerebral blood flow, aiding in the detection of early-stage cerebrovascular diseases (18, 19). Another innovative approach is the use of DTI, which quantifies the diffusion of water molecules in the brain (20, 21). DTI has proved valuable in evaluating white matter damage associated with ischemic strokes and providing insights into microstructural changes that may contribute to cognitive impairment. Moreover, molecular imaging techniques, such as PET, can provide information on specific metabolic or molecular processes in the brain (22). For instance, PET imaging with amyloid tracers can aid in the diagnosis and differentiation of vascular dementia and Alzheimer’s disease, which often coexist or share similar clinical presentations (23, 24). In short, the use of advanced imaging techniques allows for better characterization of the underlying pathology, assessment of tissue viability, selection of appropriate treatment strategies, and monitoring of treatment response. Continued research and development of novel imaging methods will further enhance our ability to diagnose, treat, and prevent cerebrovascular diseases, ultimately improving patient outcomes.

## 2 MRI

MRI is a powerful imaging modality that plays a key role in the diagnosis and assessment of cerebrovascular diseases (25). MRI provides detailed anatomical and functional information non-invasively, allowing clinicians to evaluate the extent of brain damage, identify the underlying pathology, and guide appropriate treatment strategies. In this section, we will explore various MRI techniques and their applications in cerebrovascular diseases.

### 2.1 Conventional MRI techniques

Conventional MRI sequences, including T1-weighted (T1WI), T2-weighted (T2WI), and fluid-attenuated inversion recovery (FLAIR), are routinely used in the evaluation of cerebrovascular diseases. T1-weighted images provide excellent anatomical detail, allowing visualization of the brain structures and identification of acute hemorrhage. T2-weighted and FLAIR images are sensitive to abnormalities such as edema, ischemia, and chronic hemorrhage, making them valuable in the assessment of stroke-related changes.

### 2.2 FMRI and its applications

In addition to Conventional MRI sequences, fMRI also has been used to study functional reorganization following stroke (26). It is a technique that measures and maps changes in blood oxygenation levels in the brain, allowing the assessment of functional activity. It relies on the blood oxygen level-dependent (BOLD) contrast, which is sensitive to changes in local oxygen consumption and blood flow (27). FMRI can evaluate brain activity during tasks or at rest, providing valuable information about the functional integrity of specific brain regions. Besides, it can identify areas of the brain that compensate for damaged regions and help predict motor recovery or cognitive outcomes (28–30). Furthermore, fMRI can aid in the mapping of eloquent areas of the brain, guiding surgical planning to minimize the risk of postoperative deficits (31).

### 2.3 PWI

PWI is a dynamic MRI technique that assesses cerebral blood flow (CBF) in real-time (32). PWI uses an exogenous contrast agent, such as gadolinium, to measure the transit of contrast material through the brain’s vasculature. By measuring the first pass of contrast, PWI provides quantitative information about CBF, cerebral blood volume (CBV), and mean transit time (MTT). PWI is beneficial in the evaluation of acute ischemic strokes as well (33). It can distinguish between regions of hypoperfusion and regions of completed infarction, aiding in the selection of patients who may benefit from reperfusion therapies. PWI can also identify the ischemic penumbra, a region of hypoperfused but viable tissue, which is essential for guiding treatment decisions.

### 2.4 Diffusion-weighted imaging (DWI)

DWI is a technique that measures the random motion of water molecules within the brain. In cerebrovascular diseases, DWI is highly sensitive to the early changes associated with acute ischemic strokes (34). Decreased diffusion (restricted water diffusion) on DWI images indicates cytotoxic edema, reflecting the loss of cell membrane integrity due to ischemia (35).

DWI is largely dispensable in the early diagnosis of ischemic strokes, as it can detect lesions within minutes of symptom onset. It can provide valuable information about the location, size, and severity of infarctions. Additionally, DWI can differentiate between acute and chronic lesions, helping to determine the time of stroke onset, which is essential for selecting appropriate treatment options. However, studies point out a small but significant percentage of patients with DWI-negative scan acute ischemic stroke, putting forward a clinical doctor should raise vigilance for current limitations in the diagnostic sensitivity of DWI (36).

### 2.5 DTI

DTI is a more advanced MRI technique that provides information about the microstructural organization of white matter in the brain based on anisotropic diffusion characteristics of water molecules (37). It measures the diffusion of water molecules in multiple directions and calculates the diffusion tensor, which represents the magnitude and directionality of diffusion in each voxel (38, 39). DTI has various applications in cerebrovascular diseases. It allows for the assessment of white matter integrity and provides insights into the structural changes occurring due to ischemic or hemorrhagic stroke (40). By reconstructing white matter tracts, DTI enables the study of connectivity patterns in the brain, providing valuable information about how cerebrovascular diseases affect the overall neural network (41). This can help in understanding the impact of stroke on brain connectivity and lead to a better understanding of functional deficits observed in patients (42). It allows for the identification of acute white matter injury and the evaluation of the extent and location of ischemic or hemorrhagic lesions (43). It can also provide key clues about the integrity of fiber tracts in the affected area, which is particularly important in predicting functional outcomes in stroke patients (44, 45). Long-term DTI follow-up observation is important to deepen people’s understanding of the clinical-pathological evolution process after stroke.

### 2.6 Diffusion kurtosis imaging (DKI)

DKI is another advanced MRI technique in cerebrovascular diseases because it can provide valuable insights into the microstructural changes that occur in the brain due to these conditions (46). Jensen and his colleagues proposed that the DKI technique was an extension of DTI and introduced a fourth-order tensor into the DTI imaging formula, making the minimum number of DKI scanning directions 21 (47). DKI offers several advantages in the assessment of cerebrovascular diseases compared to traditional DTI. DTI measures the diffusion of water molecules in the brain and provides information about the average diffusivity and directional fiber orientation (48). However, in regions affected by cerebrovascular diseases, there can be substantial changes in water diffusion that are not accurately captured by DTI alone (49). In contrast, DKI measures both the diffusivity and the degree of non-Gaussian diffusion within brain tissue (50). This additional information is particularly valuable for capturing changes in microstructural complexity, such as in the presence of ischemic injury, edema, or tissue degradation (51). DKI can help differentiate acute from chronic lesions, distinguish between different stages of disease severity in cerebrovascular diseases, such as stroke and small vessel disease, and monitor treatment response (49). By quantifying non-Gaussian diffusion characteristics, DKI can provide more precise and sensitive information about tissue alterations in cerebrovascular diseases. This can aid in early diagnosis, individualized treatment planning, and monitoring of therapeutic efficacy. Ultimately, DKI contributes to a better understanding of the pathophysiology and progression of cerebrovascular diseases, facilitating improved patient management and outcomes (52). Here is a short table that may explain the difference among DWI, DTI and DKI clearly (Table 1).

**Table 1:** Comparison among DWI, DTI, and DKI.

	DWI	DTI	DKI
Time of occurrence	E. O. Stejskal and J. E. Tanner in 1964	P. J. Basser et al. in 1994	J. H. Jensen et al. in 2005
Theoretical basis	Brownian motion of water molecules	Anisotropic diffusion characteristics of water molecules	The diffusion form of water molecules as a non-Gaussian distribution
Dimension	Plane	Three-dimensional	Four-order
Time for examination	1–5 min	5–15 min	5–15 min
Main parameter	Apparent diffusion coefficient, ADC	Mean diffusivity, MD; Fractional anisotropy, FA; Relative anisotropy, RA; Volume ratio, VR; etc.	Mean kurtosis, MK; Kurtosis anisotropy, KA; Axial kurtosis, AK; Radial kurtosis, RK; etc.
Application characteristics	High sensitivity and specificity in diagnosing cerebral ischemia	Tracking white matter fiber bundles in the brain	Reflecting the microstructural characteristics of brain tissue
References	(34, 53)	(37, 54, 55)	(46, 47, 50, 51)

### 2.7 Magnetic resonance angiography (MRA)

MRA is a non-invasive imaging technique that provides detailed visualization of the brain’s blood vessels. MRA uses various imaging sequences, such as time-of-flight (TOF) and contrast-enhanced MRA, to assess the arterial and venous vasculature. In cerebrovascular diseases, MRA is particularly useful in the evaluation of vascular abnormalities, such as stenosis, occlusions, aneurysms, or AVMs (56, 57). MRA can help identify the underlying cause of stroke and guide appropriate treatment options (58). It allows for preoperative planning in patients undergoing surgical interventions, such as carotid endarterectomy or aneurysm clipping.

### 2.8 Advanced MRI techniques: magnetic resonance spectroscopy, functional connectivity MRI, and intravoxel incoherent motion imaging

MRS is a technique that measures specific biochemical concentrations in the brain (59). It provides information about metabolite levels, such as N-acetyl aspartate (NAA), choline, creatine, and lactate. MRS can be used to assess the metabolic changes associated with cerebrovascular diseases, such as ischemia, hypoxia, or mitochondrial dysfunction (60). Besides, a recent study reported that in patients monitored by targeted temperature management after cardiac arrest, brain MRS–determined decrease in total NAA/creatine and an increase in lactate/creatine correlate with poor prognosis, providing Class IV evidence (61).

FcMRI is a technique that measures the temporal correlation of BOLD signals between different brain regions (62, 63). It assesses the functional connectivity networks within the brain.

IVIM imaging, first proposed by LeBihan et al. in 1986, is a technology in MRI gradually coming into view (64). IVIM can separate tissue diffusion and microvascular perfusion information, providing more accurate diffusion information to diagnostic physicians, and it has become a hot spot in recent years (65).

## 3 CT

CT is a widely used imaging modality in the diagnosis and assessment of cerebrovascular diseases. It provides rapid and detailed images of the brain, allowing clinicians to evaluate the presence, location, and extent of lesions, identify underlying vascular abnormalities, and guide appropriate treatment strategies. In this section, we will explore various CT techniques and their applications in cerebrovascular diseases.

### 3.1 Overview of CT techniques

CT imaging involves the use of X-rays and computer algorithms to generate cross-sectional images of the body. X-ray source and detectors rotate around the body of the patient, acquiring multiple X-ray images from different angles. These images are then reconstructed using specialized software, producing detailed images of the brain. Conventional CT techniques, such as non-contrast CT (NCCT) and contrast-enhanced CT (CECT), are routinely used in the evaluation of cerebrovascular diseases. NCCT images provide excellent visualization of acute hemorrhage, calcifications, and bony structures (66). CECT, on the other hand, involves the administration of an iodinated contrast agent to enhance vascular structures, aiding in the evaluation of vascular abnormalities.

### 3.2 Computed tomography angiography (CTA)

CTA is a CT technique that provides detailed visualization of the blood vessels in the brain. CTA involves the injection of an iodinated contrast agent, which highlights the blood vessels, allowing for the assessment of arterial and venous vasculature (67). In cerebrovascular diseases, CTA is particularly useful in the evaluation of vascular abnormalities, such as stenosis, occlusions, aneurysms, or AVMs. CTA can help identify the underlying cause of stroke and guide appropriate treatment options. Additionally, CTA allows for preoperative planning in patients undergoing surgical interventions, such as carotid endarterectomy or aneurysm clipping (68, 69). Besides, advanced CTA techniques, such as time-resolved CTA (4D-CTA), have improved the temporal resolution of CTA images, allowing for better assessment of dynamic vascular abnormalities and providing valuable information about the flow patterns within the blood vessels (70, 71). In an imaging study of moyamoya disease, 4D-CTA showed strong consistency and correlation with digital subtraction angiography (DSA) but was not detailed enough for the assessment of collateral circulation. 4D-CTA can serve as an alternative option for the staging and follow-up of moyamoya disease (72). Studies suggest that color-mapping of 4D-CTA can be used to detect cranial arteriovenous shunts, making color-mapping a hopeful visualization tool for assessing temporal hemodynamics in 4D-CTA (73).

### 3.3 Perfusion CT (PCT)

PCT is a dynamic CT technique that assesses CBF, cerebral blood volume (CBV), and MTT in real-time. PCT involves the injection of an iodinated contrast agent, followed by rapid acquisition of CT images, capturing the passage of contrast material through the brain’s vasculature (74). PCT is particularly useful in the evaluation of acute ischemic strokes (75, 76). It can differentiate between regions of hypoperfusion and regions of completed infarction, aiding in the selection of patients who may benefit from reperfusion therapies. PCT can also identify the ischemic penumbra, a region of hypoperfused but viable tissue, which is essential for guiding treatment decisions (77).

### 3.4 Dual-energy CT (DECT)

**Figure 1:**
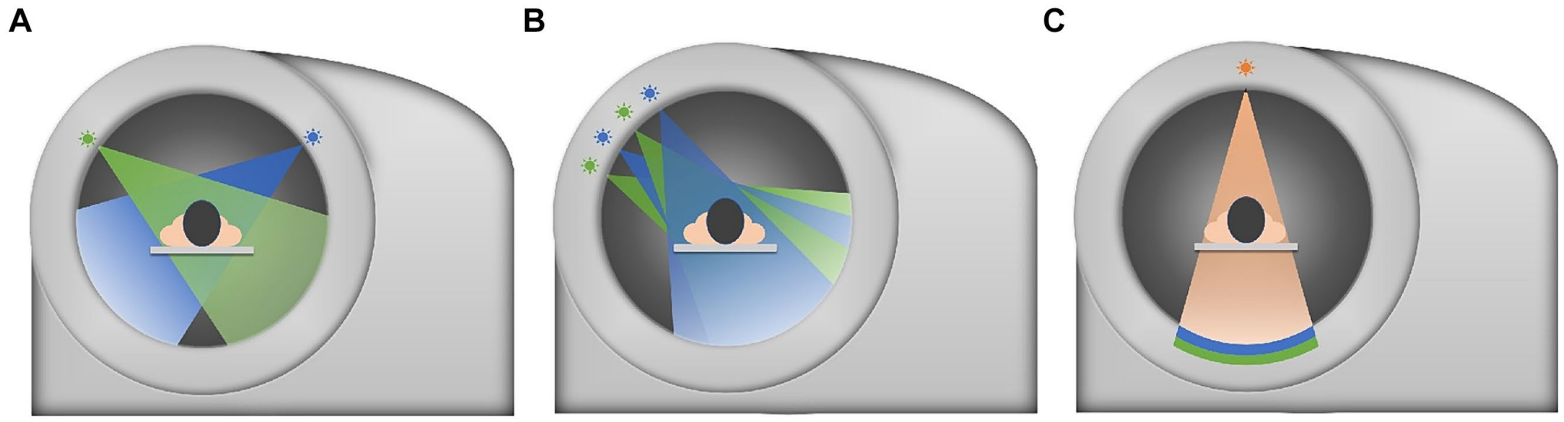
Sketch map of three main types DECT. **(A)** Dualsource-DECT: Composed of two independent x-ray tubes and two independent detectors. **(B)** Singlesource-DECT: Containing a dedicated generator that can quickly switch between low-energy and high-energy projections that are collected separately by a fast sampling-detector. **(C)** Duallayer-detector DECT: Composed of a two-layers-detector with different energy sensitivity.

DECT is an advanced CT technique that uses two different energy levels of X-rays to provide additional information about tissue composition (Figure 1) (78). With conventional CT, only one X-ray spectrum of mixed energy is used, thus losing all the energy dependence of a particular tissue. Unfortunately, for the same X-ray beam energy, if the decay of two tissues is similar (such as calcium and bone), the two tissues will be assigned the same Hounsfield unit value and it will be difficult to distinguish between them. Thus, the new technology called “dual-energy CT imaging” emerged as the times require. It can use two different energy X-ray image objects, and can accurately get the composition of the object proportion (79). By analyzing the differences in X-ray attenuation at different energy levels, DECT can distinguish between different tissue types and detect subtle changes that may not be apparent in conventional CT images (80).

In cerebrovascular diseases, DECT has shown promise in the evaluation of cerebral hemorrhage, especially in differentiating between active bleeding and hemorrhagic transformation of an ischemic stroke (81). Although both cerebral hemorrhage and contrast agent extravasation present high-density shadows, they can still be distinguished by DECT according to water-based and iodine-based maps, which is hard for routine CT scans as they both present high-density shadows. DECT can also aid in differentiating between calcium and iodine in vascular structures, providing valuable information about vessel wall calcifications and enhancing the assessment of vascular abnormalities (82, 83).

## 4 PET

### 4.1 Principles and techniques of PET imaging

PET is a nuclear medicine imaging technique that provides functional information about various physiological processes in the body. PET imaging involves the administration of radiotracers, labeled with positron-emitting isotopes, into the patient’s body. These radiotracers undergo radioactive decay and emit positrons, which then interact with surrounding electrons, resulting in the emission of two gamma rays (84). These gamma rays are detected by a circular array of detectors surrounding the patient, allowing for the reconstruction of images (3).

PET imaging utilizes several different radiotracers to target specific physiological processes. For cerebrovascular diseases, commonly used radiotracers include fluorodeoxyglucose (FDG) (85), which measures glucose metabolism, and oxygen-15 labeled water (H2^15O), which measures regional cerebral blood flow (rCBF) (86). Other tracers such as carbon-11 labeled Pittsburgh compound B (PiB) and fluorine-18 labeled florbetapir have been used to evaluate cerebral amyloid deposition in conditions like Alzheimer’s disease (87, 88).

### 4.2 Applications of PET in cerebrovascular diseases

PET imaging has a wide range of applications in cerebrovascular diseases. One of the primary uses is the evaluation of cerebral blood flow and metabolism. PET can provide valuable information about the extent and severity of cerebral ischemia in patients with acute stroke or chronic cerebrovascular disease (45). It can also be used to assess the viability of brain tissue in regions at risk of infarction before interventional procedures such as carotid endarterectomy. It can also be utilized for the detection and characterization of cerebral amyloid deposition in disorders like Alzheimer’s disease (89, 90). The use of specific amyloid-targeting tracers allows for the quantification of amyloid plaques in the brain, aiding in diagnosis and monitoring disease progression (91, 92). Furthermore, PET imaging can help in the evaluation of other cerebrovascular disorders such as cerebral vasculitis and moyamoya disease. It can provide information regarding the inflammatory activity, regional blood flow, and metabolism in affected regions, aiding in the diagnosis and treatment planning (93).

### 4.3 Novel PET tracers and their diagnostic potential

Advancements in PET technology have led to the development of novel radiotracers with improved diagnostic potential (94). For example, flortaucipir, a tau-specific PET tracer, can be used to visualize and quantify tau pathology in neurodegenerative diseases such as Alzheimer’s disease (95, 96). This allows for a better understanding of the underlying disease processes and potentially enables the development of targeted therapies. Other emerging PET tracers include those targeting specific neurotransmitter receptors or transporters, which can provide insights into neurochemical imbalances in cerebrovascular diseases (97). For instance, PET tracers targeting the dopamine receptors can help evaluate the presence and severity of vascular parkinsonism (98, 99).

### 4.4 PET-CT and PET-MRI hybrid imaging

PET imaging can be combined with other imaging techniques such as CT or MRI to provide additional anatomical and functional information. Most PET scanners are actually PET/CT scanners that acquire a low-dose CT used for attenuation correction (compensation for signal loss due to structurally dense areas such as bone) during reconstruction of the PET images. This combination allows for the accurate localization of PET findings within the brain and facilitates the identification of potential underlying causes of cerebrovascular diseases, such as vascular stenosis or aneurysms (100). This fusion of modalities improves diagnostic accuracy and aids in treatment planning.

PET/MRI scanners have been slowly growing in popularity; these replace the low-dose CT with MRI-and artificial intelligence-based substitutes to eliminate the CT’s dose of radiation to the participant (101). However, achieving CT-like bone contrast with MRI is not straightforward (102), and in neurodegeneration research studies PET/ CT scanners still far outnumber PET/MRI scanners.

## 5 Single-photon emission computed tomography (SPECT)

SPECT has a pivotal role in the field of diagnosing and managing cerebrovascular diseases. It is revered for being a non-invasive technique and insightful images outlining cerebral blood flow and brain function. SPECT achieves its precise imaging by using radioactive tracers, which can penetrate the blood–brain barrier and remain in the brain cells for a long time for imaging. These tracers emit gamma rays, which the rotating camera around the patient captures. By incorporating images from various angles, SPECT effectively creates a three-dimensional representation of the distribution of the radiotracer. The amount of radioactive drugs stored in the brain is directly proportional to blood flow, so the radioactive distribution in brain tissue caught by SPECT can reflect rCBF and functional status (103, 104). For instance, it can lucidly distinguish between ischemic stroke and hemorrhagic stroke by spotlighting regions of reduced blood flow, which helps in identifying regions with compromised blood circulation in the brain, thereby indicating potentially pathologic areas. Compared with CT and MRI, SPECT has a higher sensitivity to blood flow and thus a higher positive diagnostic rate for transient ischemic attacks (TIA) and the hyperacute phase of cerebral infarction, which is of great significance for early detection of chronic hypoperfusion in TIA, prediction and prevention of cerebral infarction, and improvement of prognosis (105, 106). Besides, it can detect abnormalities in brain function, aiding in the diagnosis of diseases such as Alzheimer’s, Parkinson’s, and epilepsy (107). In summary, as technology continues to advance, SPECT imaging is expected to play an increasingly critical role in the field of neurology, contributing to further advancements in our understanding and treatment of cerebrovascular diseases.

## 6 DSA

DSA is the classic gold-standard imaging method for diagnosis in acute cerebrovascular diseases. It is of diagnostic and monitoring importance in cranial vascular stenosis or occlusion, aneurysms, arteriovenous malformations and moyamoya disease, as well as evaluation of the relationship between blood supply of intracranial lesions and adjacent blood vessels, etiological exploration of spontaneous intracranial hematoma and subarachnoid hemorrhage. The basic content of DSA is to perform two imaging tasks by applying X-ray examination technology and computer programs, subtracting two frames of images of the same part of the human body, in order to obtain its different parts. With the development of technology, the role of DSA is becoming increasingly prominent in directing the clinical treatment and judging the prognosis, rather than limited to diagnosis in the neuro-interventional field (108). In the endovascular treatment of acute ischemic stroke patients, DSA is commonly used to guide endovascular treatment, in order to identify thrombus retrieval and improve the therapeutic effect of stent retrieval thrombectomy. DSA has been shown to guide the treatment in patients with large vessel cerebral vasospasm, delayed cerebral ischemia in subarachnoid hemorrhage and aneurysms, such as endovascular surgery for symptomatic vasospasm treatment by balloon angioplasty. Besides, increasing clinical research illustrated the prognostic value in patients with ischemia, intracranial aneurysm, moyamoya disease and vasculitis according to different degrees, sites, collaterals or recanalization and so on (109, 110). For example, Liu and colleagues showed that the appearance of lateral lenticulostriate arteries before mechanical thrombectomy detected by DSA may help predict a good prognosis in patients with acute middle cerebral artery occlusion in M1 segment (111). While considering the radiation exposure and potential risks of intervention operations, DSA is not commonly used as the preferred method for vascular assessment and surveillance.

## 7 Challenges and limitations of novel imaging methods

### 7.1 Technical limitations

Novel imaging methods often come with technical limitations that can impact their utility and reliability. For example, some techniques may have lower spatial resolution, limiting their ability to detect small lesions or subtle changes in tissue. Similarly, certain imaging methods may have longer scan times, which can be challenging for patients who are unable to remain still for extended periods.

Furthermore, novel imaging methods may require specialized equipment or expertise, making them less accessible in certain healthcare settings or regions with limited resources. The availability of trained personnel and maintenance of complex imaging systems can also pose challenges.

Another technical limitation is the potential for artifacts or image distortions, which may arise due to patient motion, metallic implants, or other factors. These artifacts can compromise the accuracy and interpretation of imaging findings, requiring careful consideration and additional measures to mitigate their impact (112).

### 7.2 Interpretation challenges

Interpreting data from novel imaging methods can be complex and subjective, presenting challenges to clinicians and researchers. The interpretation of images may require specialized knowledge and expertise, and the reliability of these interpretations can vary among individuals.

Moreover, the lack of standardized protocols and established reference values can impede the consistency and comparability of findings across different healthcare institutions and studies (113, 114). This makes it challenging to establish reliable diagnostic criteria or thresholds for abnormality in novel imaging methods.

Additionally, the integration of imaging findings with clinical data, including patient history and other diagnostic tests, is necessary for accurate interpretation. However, incorporating this multimodal information can be challenging, requiring expertise in multiple domains and potentially leading to increased variability in interpretation.

### 7.3 Cost-effectiveness considerations

The adoption of novel imaging methods can be hindered by cost-related factors. Advanced imaging techniques often require specialized equipment, software, and infrastructure, which can be expensive to acquire, maintain, and upgrade (115). The high costs associated with these technologies may limit their availability to only certain healthcare facilities or regions, preventing widespread access to these diagnostic tools.

Moreover, the cost-effectiveness of novel imaging methods must be carefully evaluated, considering the potential benefits and impact on patient outcomes. Assessing the cost-effectiveness of these methods requires robust evidence of their diagnostic accuracy, potential impact on clinical decision-making, and improvements in patient outcomes. Economic evaluations are crucial to determine whether the benefits outweigh the costs and can guide policy decisions regarding reimbursement and resource allocation (116).

### 7.4 Ethical and regulatory concerns

The use of novel imaging methods raises ethical and regulatory considerations. Ensuring patient safety and data privacy is paramount when implementing these techniques (117). Additionally, the ethical implications of novel imaging methods should be carefully considered. For instance, the potential for incidental findings that are unrelated to the initial clinical question may arise. Decisions on how to manage and communicate these incidental findings to patients require careful ethical considerations (118). Moreover, the development and validation of novel imaging methods may involve research on human subjects. Ethical review boards and regulatory bodies play a central role in ensuring that research involving these techniques is conducted with proper informed consent, minimizes risks, and adheres to ethical principles (119).

## 8 Future directions and conclusion

### 8.1 Potential advancements in imaging technology

The field of medical imaging is continuously evolving, and several potential advancements hold promise for the future of cerebrovascular disease management. Improved imaging techniques with higher spatial resolution and faster acquisition times may become available, allowing for more accurate and efficient diagnosis and monitoring of cerebrovascular diseases (120).

Advancements in hardware and software may lead to the development of more portable and accessible imaging devices, expanding their use in different healthcare settings, including remote areas and low-resource settings. Additionally, the integration of imaging technology with other modalities, such as genomics or proteomics, may provide comprehensive and personalized insights into the underlying pathophysiology of cerebrovascular diseases.

### 8.2 Role of AI in the future of cerebrovascular disease management

Artificial intelligence (AI) is poised to play a significant role in the future of cerebrovascular disease management (121, 122). Machine learning and deep learning algorithms can learn from vast datasets to identify patterns and predict outcomes (123). These algorithms can assist in the automated analysis of imaging data, allowing for faster and more consistent interpretation (124). Moreover, the integration of AI with imaging techniques can enable the development of predictive models to identify high-risk patients, tailor treatment plans, and monitor treatment response (125).

The integration of artificial intelligence algorithms with imaging technologies has the potential to significantly improve the diagnosis and assessment of cerebrovascular diseases (126). AI algorithms can analyze large amounts of imaging data, identify subtle abnormalities, and assist in the interpretation of images (127). AI algorithms can be trained to automatically detect and segment lesions, such as ischemic infarctions, hemorrhages, or vascular abnormalities, from images. Newer algorithms using machine learning techniques are promising, but these algorithms require high-standard training datasets to optimize performance. J.E. Soun and colleagues summarized and compared several publicly available imaging datasets required for machine learning algorithm optimization execution, including ATLAS (Anatomical Tracings of Lesions After Stroke), CQ500 dataset (which was provided by the Center for Advanced Research in Imaging, Neurosciences and Genomics, New Delhi, India), Radiology Society of North America Brain Hemorrhage CT Dataset, ASFNR (American Society of Functional Neuroradiology) for stroke and ISLES (Ischemic Stroke Lesion Segmentation) for ischemic stroke (128–131). Recently, commercial software platforms, including Brainomix, RapidAI, *Viz.*ai, Avicenna.AI and Aidoc, have been implemented in various stroke centers, utilizing AI to treat ischemic and hemorrhagic stroke (132). They can aid radiologists in the quantification and characterization of lesions, providing more accurate and reproducible measurements (133). Additionally, AI algorithms can help predict patient outcomes, such as the risk of recurrent strokes or response to treatment, based on imaging findings and clinical data (134, 135). While, a study conducted by Voter AF and colleagues showed unexpectedly lower sensitivity and positive predictive values for Aidoc in diagnosing intracranial hemorrhage, which has raised concerns about the generalizability of these commercial AI tools (136).

Furthermore, AI algorithms can assist in the triaging of patients, prioritizing urgent cases, and expediting the diagnosis and management of cerebrovascular diseases. They can flag critical findings, notify clinicians for immediate action, improve patient care, and reduce the time to treatment.

Machine learning and deep learning algorithms can learn from vast datasets to identify patterns and predict outcomes. These algorithms can assist in the automated analysis of imaging data, allowing for faster and more consistent interpretation. Moreover, the integration of AI with imaging techniques can enable the development of predictive models to identify high-risk patients, tailor treatment plans, and monitor treatment response.

### 8.3 Integration of multimodal imaging techniques

**Figure 2:**
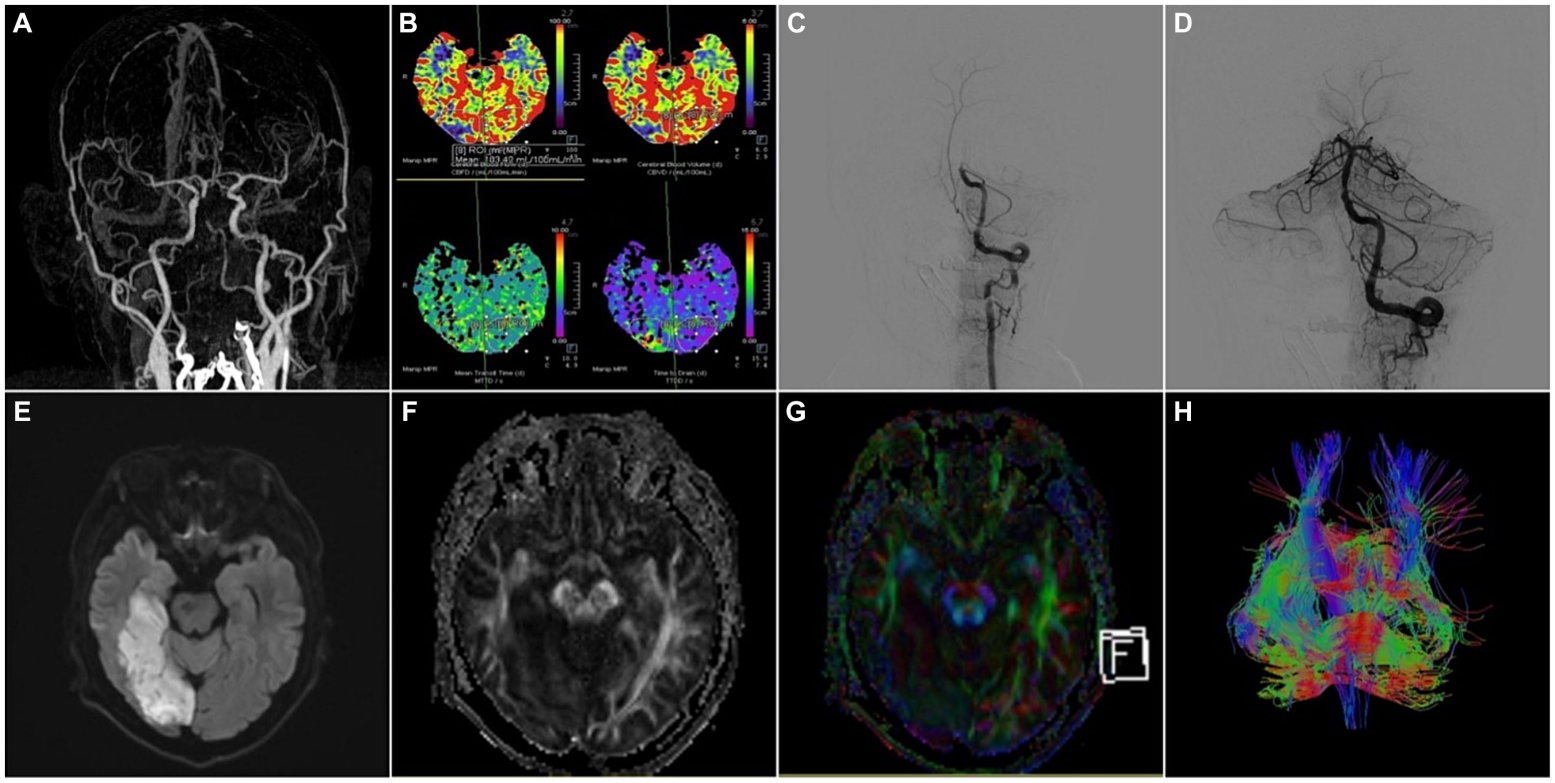
A 52-year-old male patient was admitted to the hospital with “weakness of left limb for 5 h” and was treated with intravenous thrombolysis by alteplase combined with arterial thrombectomy. **(A)** CTA-MIP: occlusion of the basilar artery; **(B)** PCT: the picture contained four important parameters of PCT like CBF, CBV, MTT and TTD, which showed the value of CBF, CBV became lower and the value of MTT, TTD became longer in the right occipital lobe compared to the left, that meant hypoperfusion in the basilar artery supply area; **(C,D)** DSA: occlusion of the basilar artery was demonstrated by left vertebral artery angiography, and the basilar artery was recanalized with moderate proximal stenosis after thrombectomy, the left posterior cerebral artery was patent, and the distal right posterior cerebral artery did not have significant visualization; **(E)** MRI-T2WI, **(F)** MRI-DTI-FA, **(G)** MRI-DTI-colorful FA, **(H)** MRI-DTI-fiber tractography: 4 days after thrombectomy, T2WI showed the right temporo-occipital lobe had a patchy infarct focus, and DTI demonstrated the right tractus pyramidalis were thinner than those on the left side.

**Figure 3:**
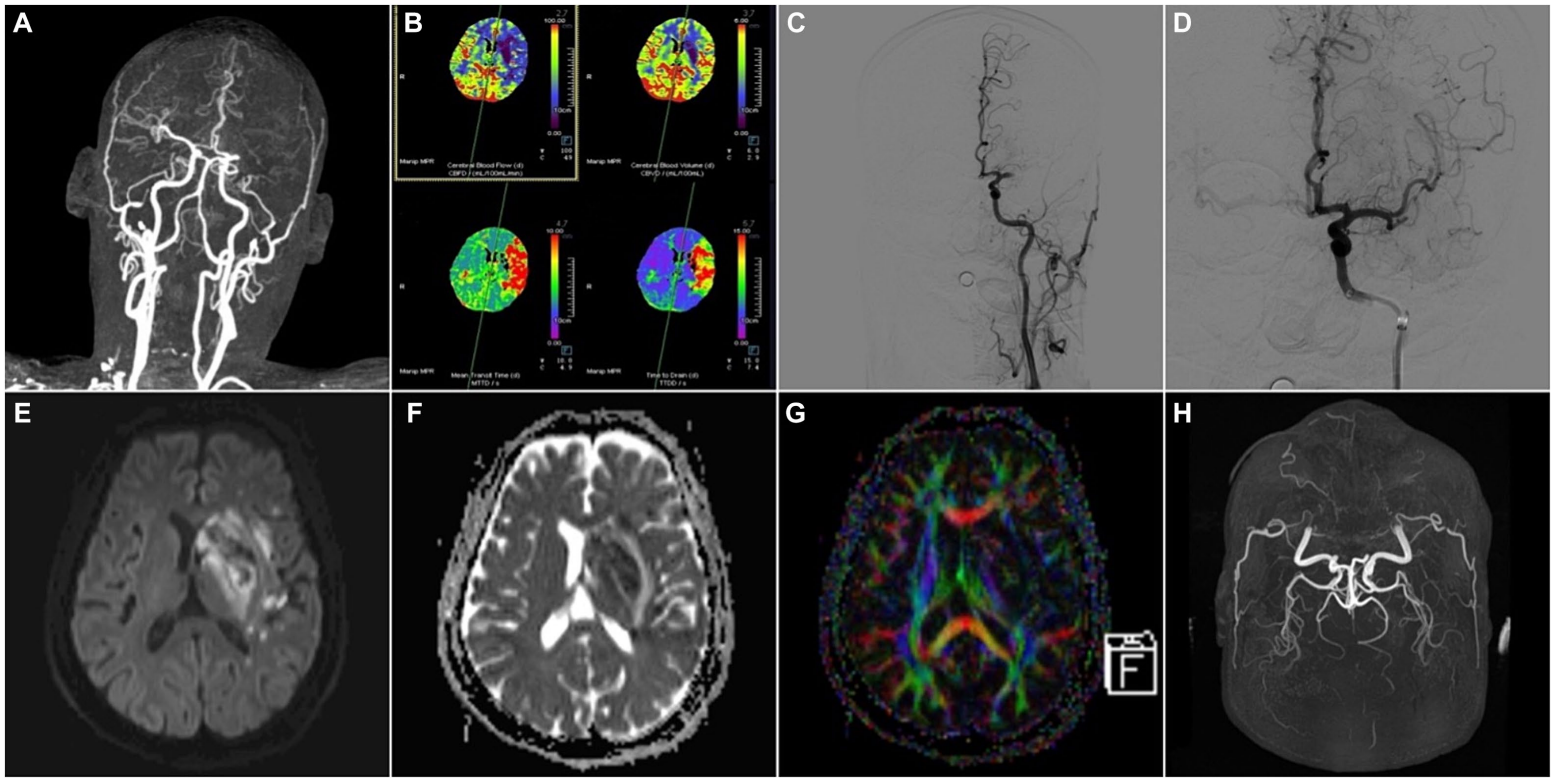
A 59-year-old male patient was admitted to the hospital with “weakness of right limb for 13 h” and was treated with arterial thrombectomy. **(A)** CTA-MIP: occlusion of the left middle cerebral artery; **(B)** PCT: the value of CBF, CBV became lower and the value of MTT, TTD became longer in the left middle cerebral artery supply area compared to the right, which meant hypoperfusion in the left middle cerebral artery supply area; **(C,D)** DSA: angiography suggested occlusion of the left middle cerebral artery, and after thrombectomy, re-angiography showed that the main trunk of the left middle cerebral artery was patent, with some thrombus escaping to the distal branches; **(E)** MRI-DWI, **(F)** MRI-ADC, **(G)** MRI-DTI-colorful FA: 3 days after thrombectomy, DWI and ADC demonstrated multiple infarct foci in the blood-supplying area of the left middle cerebral artery, and a few hemorrhagic transformed foci in the basal ganglia area, DTI showed the left nerve fiber bundles were thinner than those on the right side; **(H)** MRA-TOF: 4 days after thrombectomy, the left middle cerebral artery was still patent.

The integration of multiple imaging modalities can provide a more comprehensive and holistic assessment of cerebrovascular diseases. Combining data from different imaging techniques, such as PET, DTI, MRI, or CT, can provide complementary information about various aspects of the disease, including vascular and metabolic changes, tissue integrity, and functional connectivity (Figures 2, 3) (137).

Multimodal imaging can help overcome the limitations of individual imaging techniques, enabling more accurate diagnosis, treatment planning, and monitoring of cerebrovascular diseases. However, challenges related to data fusion, standardization, and interpretation need to be addressed to fully leverage the potential of multimodal imaging.

### 8.4 Personalized medicine approaches

The future of cerebrovascular disease management is moving toward personalized medicine approaches. By integrating imaging data, genetic information, clinical data, and other biomarkers, it will be possible to tailor treatment strategies to individual patients.

Personalized medicine approaches can help identify patients at high risk of cerebrovascular diseases, optimize treatment selection, predict treatment response, and monitor disease progression. These approaches can lead to more targeted and effective interventions, improving patient outcomes and reducing the burden of cerebrovascular diseases.

## 9 Discussion and prospection

In recent years, cerebrovascular imaging technology has developed rapidly. In China, a potential developing country, basic technologies such as CTA, MRA and DSA have been widely applied in grassroots hospitals, revolutionizing the overall level of clinical working abilities in cerebrovascular diseases. The same situation applies worldwide, from traditional methods like CT and MRI to advanced techniques such as PET and DTI, these imaging modalities provide valuable insights into the underlying pathology, connectivity, and function of the brain. Still, there are some technologies under intense research that have great potential for clinical application. For instance, a prospective study conducted by Ma and colleagues cleared up the threshold of multidelay arterial spin labeling (MDASL) in penumbra volume of acute ischemic stroke, which has brought this technology closer to large-scale clinical applications (138). Moreover, the future of cerebrovascular disease management holds great promise with potential advancements in imaging technology, the integration of AI algorithms, the utilization of multimodal imaging techniques, and the adoption of personalized medicine approaches. These developments will enhance diagnostic accuracy, improve treatment planning, and enable better patient outcomes. However, challenges and limitations must be addressed, including technical limitations, interpretation challenges, cost-effectiveness considerations, and ethical and regulatory concerns. By overcoming these challenges, the full potential of novel imaging methods can be harnessed to benefit patients with cerebrovascular diseases and advance our understanding of these complex conditions.

In conclusion, the ongoing advancements in imaging technology and the integration of AI algorithms, multimodal imaging, and personalized medicine approaches hold great promise for the future of cerebrovascular disease management. Continued research, technological developments, and collaborations are warranted to realize the full potential of these advancements and improve clinical applications in the field of cerebrovascular diseases.

## Author contributions

FL: Writing – original draft. YiY: Writing – review & editing. YuY: Writing – review & editing. RR: Conceptualization, Writing – review & editing. YH: Conceptualization, Writing – review & editing. BZ: Visualization, Writing – review & editing.

## Figures and Tables

**Table:** 

ADC	Apparent diffusion coefficient
AI	Artificial intelligence
AVMs	Arteriovenous malformations
BOLD	Blood oxygen level-dependent
CBF	Cerebral blood flow
CBV	Cerebral blood volume
CECT	Contrast-enhanced CT
CT	Computed Tomography
CTA	Computed tomography angiography
DECT	Dual-energy CT
DKI	Diffusion kurtosis imaging
DSA	Digital subtraction angiography
DTI	Diffusion Tensor Imaging
DWI	Diffusion-weighted imaging
FA	Fractional anisotropy
fcMRI	Functional connectivity MRI
FDG	Fluorodeoxyglucose
FLAIR	Fluid-attenuated inversion recovery
fMRI	Functional MRI
IVIM	Intravoxel incoherent motion
MDASL	Multidelay arterial spin labeling
MIP	Maximum intensity projection
MRA	Magnetic resonance angiography
MRI	Magnetic Resonance Imaging
MRS	Magnetic resonance spectroscopy
MTT	Mean transit time
NAA	N-acetyl aspartate
NCCT	Non-contrast CT
PCT	Perfusion CT
PET	Positron Emission Tomography
PiB	Pittsburgh compound B
PTC	Perfusion computed tomography
PWI	Perfusion-weighted imaging
rCBF	Regional cerebral blood flow
SPECT	Single-Photon Emission Computed Tomography
T1WI	T1-weighted
T2WI	T2-weighted
TIA	Transient ischemic attacks
TOF	Time of flight
TTD	Time to drain
